# The Use of Hyperbaric Oxygen Therapy in the Management of Severe Radiation-Induced Hemorrhagic Gastritis in a Pediatric Patient

**DOI:** 10.1097/PG9.0000000000000144

**Published:** 2021-12-03

**Authors:** Erica Rabinovich, Kelly Johnson-Arbor, Mariastella Serrano, Catherine Chao, Michael J. Eblan, Avani D. Rao, Michael Terao, Jeffrey Toretsky, Susmita Sarangi, Carly Varela, Dahye Hong

**Affiliations:** From the * MedStar Georgetown University Hospital, Washington, DC; † INOVA Children’s Hospital, Falls Church, VA.

**Keywords:** radiotherapy, hyperbaric oxygenation, pediatrics, rhabdomyosarcoma, anemia

## Abstract

Radiation-induced hemorrhagic gastritis is a serious and rare complication of radiation therapy. Optimal therapies in the pediatric population are not well established. We report a 2-year-old female diagnosed with rhabdomyosarcoma who developed hemorrhagic gastritis following chemotherapy and radiation therapy. The patient presented with acute onset anemia, hematemesis, and melena. Endoscopies revealed circumferential ulceration at the pylorus with spontaneous oozing that failed to respond effectively with multimodal medical and endoscopic therapies. Following hemodynamic stabilization, the patient was treated with hyperbaric oxygen therapy with excellent clinical response of the bleeding. Further research on the benefit of hyperbaric oxygen therapy is warranted to determine if this treatment can reduce the incidence of gastrointestinal complications in patients who have received radiation therapy.

## INTRODUCTION

Radiation-induced hemorrhagic gastritis (RIHG) is a serious and rare complication of abdominal radiation therapy, characterized by acute vasculopathy progressing to obliterative endarteritis, vasculitis, and endothelial proliferation. Severe RIHG can result in life-threatening bleeding secondary to ischemia and ulceration of the gastrointestinal mucosa.^1^ While cases are reported that document successful management of RIHG via various modalities, the optimal treatment, especially in the pediatric population, has not been established. We report a pediatric patient who developed severe and refractory RIHG with bleeding and anemia that resolved following hyperbaric oxygen therapy (HBOT).

## CASE REPORT

A 2-year-old female diagnosed with retroperitoneal rhabdomyosarcoma was treated with chemotherapy and proton beam radiation therapy (5040 cGy in 28 fractions). Proton therapy was delivered with beams oriented to optimally spare the patient’s kidneys, liver, heart, and small bowel. As the tumor’s bulky anatomy compressed the distal stomach and pylorus anteriorly against the abdominal wall, the distal stomach and pylorus received almost circumferential radiation to the total dose of 5040 cGy.

Three months after completing radiotherapy, when she would have been expected to have normal blood counts, she presented with pallor and anemia (hemoglobin 5.2g/dL; normal range 10.2–12.7 g/dL). She was transfused with packed red blood cells (PRBCs) but required admission 3 days later due to hematemesis, melena, and a drop in hemoglobin to 2.5 g/dL. An upper gastrointestinal endoscopy revealed circumferential ulceration at the peripyloric region with spontaneous oozing, and argon plasma coagulation (APC) therapy was performed. A clinical diagnosis of RIHG was established, and treatment with pantoprazole, sucralfate and octreotide infusion (2 µg/kg/h with titration to a maximum of 10 µg/kg/h over the next several days) was initiated. Repeat endoscopy revealed similar findings, and injection with epinephrine was performed. The patient continued to be transfusion-dependent despite these modalities; she did not have any additional identifiable comorbidities that may have increased her risk for gastrointestinal bleeding (such as coagulopathy, liver disease, or portal hypertension). Before considering surgical intervention for subtotal gastrectomy, she was transferred to our hospital for HBOT as a final potential medical therapy.

**FIGURE 1.**
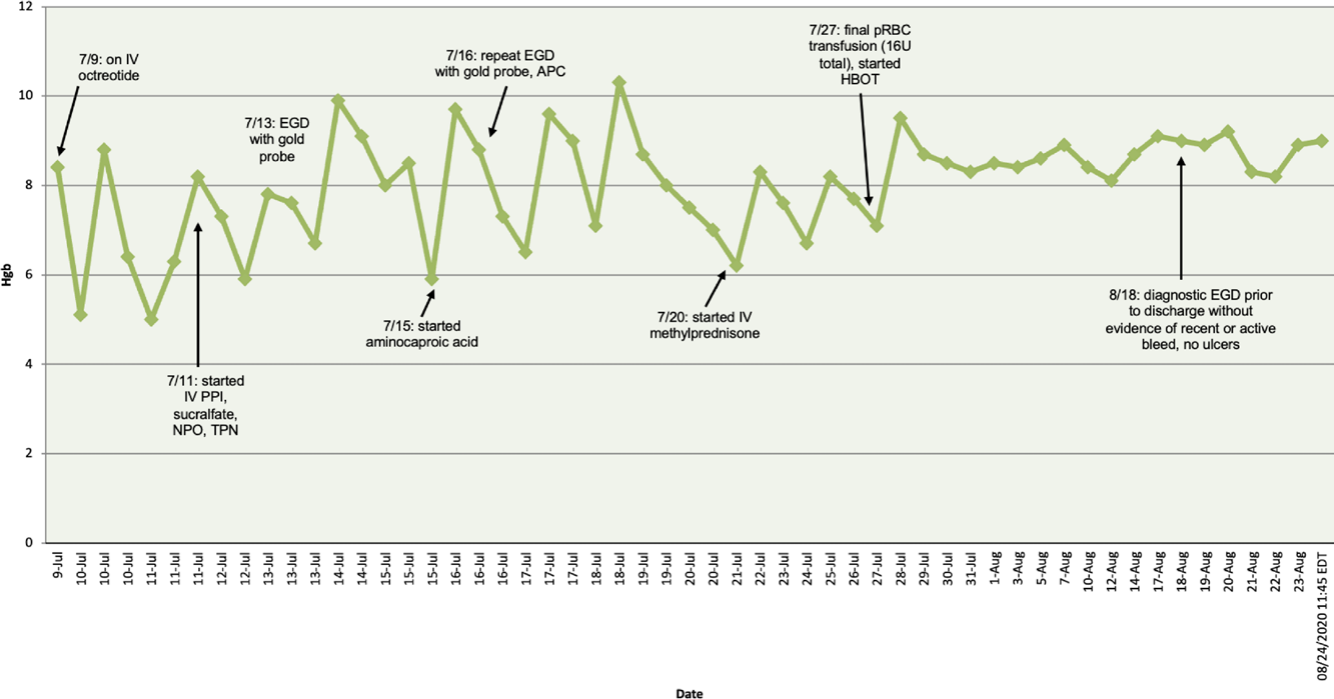
Hemoglobin curve over time. Date is on the x-axis, and hemoglobin level on the y-axis. Interventions by date are marked with arrows corresponding to date. 16U = 16 units; APC = argon plasma coagulation; EGD = esophagogastroduodenoscopy; HBOT = hyperbaric oxygen therapy; Hgb = hemoglobin; IV = intravenous NPO = nothing by mouth; PPI = proton pump inhibitor; pRBC = packed red blood cells; TPN = total peripheral nutrition.

**FIGURE 2.**
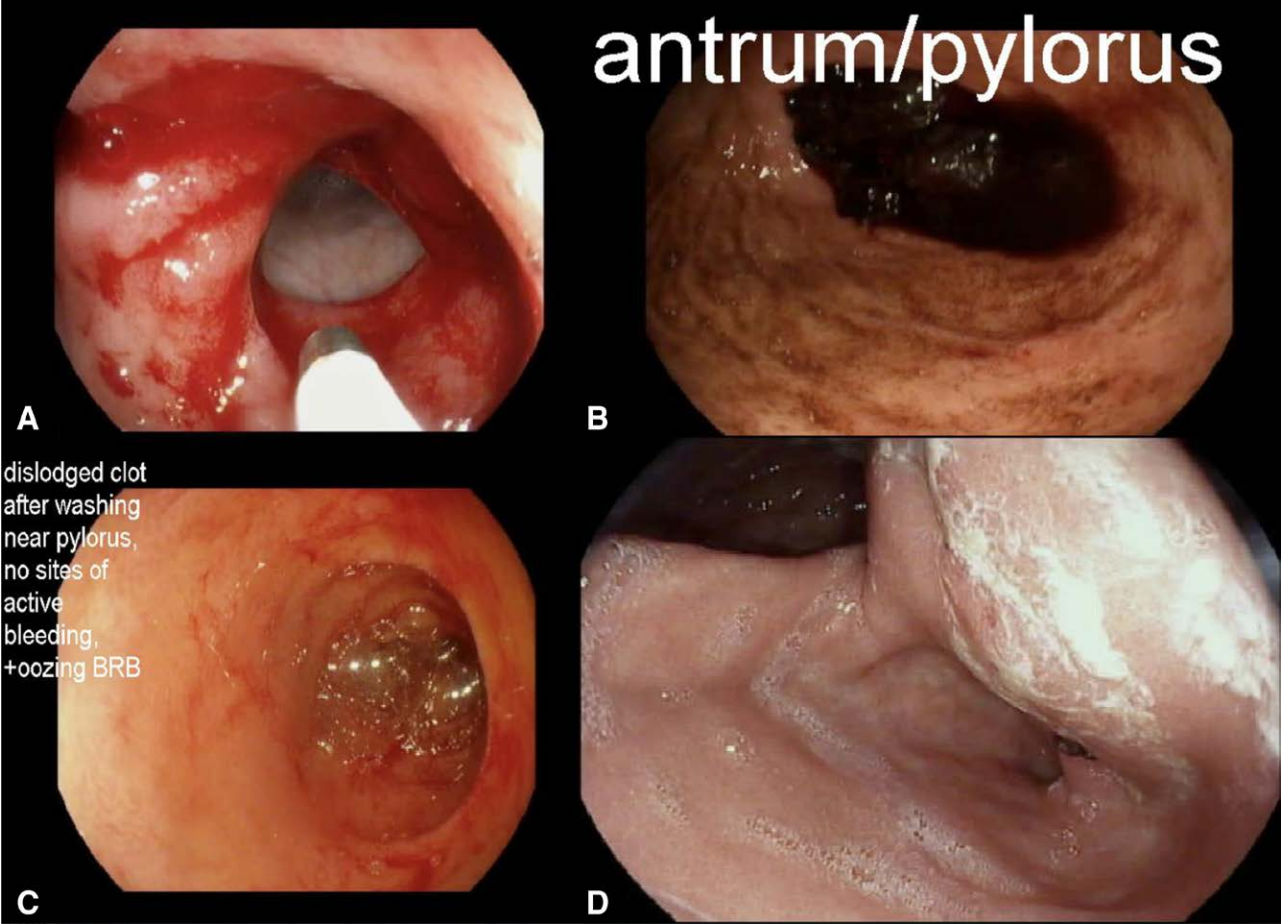
Endoscopic visualization of stomach throughout hospital course. A) Antrum of stomach with red and old blood visualized in the fundus and minor curvature. Mucosa of pylorus and prepyloric area friable, diffusely eroded, erythematous with oozing but no specific point of bleeding (July 13, 2020). B) Clot in the pylorus (July 18, 2020). C) Dislodged clot after washing near pylorus. Oozing bright red blood visualized. No site of active bleeding (July 18, 2020). D) No concerns for bleeding (August 18, 2020).

Upon admission, the medical therapies were continued that were begun at the referring hospital (Fig. 1). Bleeding, with a significant drop in hemoglobin, occurred shortly after transfer. Esophagogastroduodenoscopy was repeated using APC and contact thermal bipolar gold probe cauterization (Fig. 2).

After 18 days of hospitalization, the patient was hemodynamically stable for HBOT initiation. Bilateral tympanostomy tubes were placed, and the patient received 25 daily hyperbaric treatments to a depth of 2.0 absolute atmospheres (ATAs) in a monoplace hyperbaric chamber. Following HBOT initiation, the hemoglobin level normalized after 8 days without additional PRBC transfusion. Gastrointestinal bleeding did not recur, based on a stable hemoglobin without further transfusion and confirmed on follow-up endoscopy. She was discharged home after 7 weeks of hospitalization. The patient remained well for 2 months; however, she developed progressive disease and died 14 months after discharge. She did not have recurrence in gastric bleeding before her death.

## DISCUSSION

Radiation-induced injury to the gastrointestinal tract rarely involves the stomach.^1^ RIHG can be a serious complication of radiation therapy when orientation of beams is directed at the stomach or, as was the case in the patient described above, when the stomach is within the high-dose field of radiation. Few published reports are available concerning the management of RIHG in the pediatric population. The initial medical management of significant gastrointestinal bleeding is highlighted in Table 1.^2–5^.

**TABLE 1.** Medical management of significant gastrointestinal bleeding

Treatment modality	Rationale
Fluid resuscitation, blood products	Encourage hemodynamic stability, improve volume status
Vasopressors	Encourage hemodynamic stability, systemic perfusion
Vasopressin	Decrease splanchnic blood flow
Proton pump inhibitors, Histamine 2 receptor antagonists	Suppressing gastric acid production
Somatostatin analog	Decreasing portal blood flow
Corticosteroids	Decrease radiation induced inflammation
Aminocaproic acid	Clot stabilization to allow for mucosal healing
Sucralfate	Establish protective barrier in the gastric mucosa

First column describes the treatment modality, and the second column describes the rationale.

During HBOT, patients breathe 100% oxygen (O_2_) while enclosed in a pressurized chamber. HBOT is an accepted treatment for patients with radiation-induced skin or soft-tissue necrosis. The mechanism of action of HBOT is increased systemic arterial oxygenation, which may exceed 2000 mm Hg. In the presence of hypoxic wounds (including tissue rendered hypovascular due to previous radiation therapy), enhanced arterial O2 concentrations stimulate increased intracellular reactive species of oxygen and nitrogen, ultimately leading to tissue neovascularization and enhanced healing. HBOT is performed in a single- or multiple-person hyperbaric chamber, generally to a depth of 2–3 ATA.^6^ In patients with radiation-induced soft-tissue necrosis, HBO-induced neovascularization commonly reaches a plateau effect after approximately 20 treatments, consistent with the clinical improvement and cessation of gastrointestinal bleeding noted in our case after 25 treatments.^7^ HBOT is infrequently reported as a therapeutic option for RIHG.^1,8,9^ Banerjee et al^1^ reported an adult patient with refractory radiation-induced gastritis who received 30 HBOT sessions, after which melena stopped, and the patient did not require further transfusion.

Adverse events associated with HBOT include middle ear barotrauma, oxygen toxicity, and confinement anxiety. These risks can often be mitigated with careful attention to treatment depth, patient selection, and safety assessment. Since the patient’s young age placed her at an increased risk for middle ear barotrauma, bilateral tympanostomy tubes were placed before her first HBOT treatment. A treatment depth of 2.0 ATA was utilized to reduce her risk of central nervous system oxygen toxicity. To reduce possible confinement anxiety, the patient watched age-appropriate movies, Child Life services were provided, and one parent remained adjacent to the hyperbaric chamber throughout the treatment.

Our patient experienced a favorable short-term outcome after receiving HBOT for severe RIHG. As this represents a single case report, the overall utility of the use of HBOT in patients who have bleeding refractory to medical and endoscopic management is unclear. Since life-threatening bleeding may occur in patients with RIHG, it is reasonable to consider HBOT as an adjunctive treatment in patients with refractory RIHG. Prospective randomized trials of HBOT in patients with RIHG are an ideal way to assess the effectiveness of this treatment in this patient population, although this is unlikely to be feasible given the rarity of this clinical condition. Additionally, while HBOT is available in approximately 1300 medical facilities in the United States, not all HBOT locations are capable of treating pediatric patients.^10^ Fortunately, in the United States, major insurance carriers consider HBOT to be a medically necessary and reimbursable treatment for patients with delayed radiation injuries.

In conclusion, early consideration of HBOT can be considered in patients with refractory RIHG. Further research on the benefit of HBOT in patients with RIHG is warranted to determine if this treatment can reduce gastrointestinal complications in patients who have received radiation therapy.

## ACKNOWLEDGMENT

E.R., K.J.-A., M.S., M.T., J.T., S.S., C.V., and D.H. conceived of the case report, E.R. wrote the first draft, and C.C., M.J.E., A.D.R., K.J.-A., M.S., M.T., J.T., S.S., C.V., and D.H. edited the draft. All authors read and approved the submitted version.
